# Antagonistic effects of biological invasion and environmental warming on detritus processing in freshwater ecosystems

**DOI:** 10.1007/s00442-016-3796-x

**Published:** 2016-12-24

**Authors:** Daniel Kenna, William N. W. Fincham, Alison M. Dunn, Lee E. Brown, Christopher Hassall

**Affiliations:** 1 School of Biology and water@leeds, University of Leeds, Leeds, UK; 2 School of Geography and water@leeds, University of Leeds, Leeds, UK

**Keywords:** Leaf-litter, Amphipod, Climate change, Resource processing, Temperature

## Abstract

Global biodiversity is threatened by multiple anthropogenic stressors but little is known about the combined effects of environmental warming and invasive species on ecosystem functioning. We quantified thermal preferences and then compared leaf-litter processing rates at eight different temperatures (5.0–22.5 °C) by the invasive freshwater crustacean *Dikerogammarus villosus* and the Great Britain native *Gammarus pulex* at a range of body sizes. *D. villosus* preferred warmer temperatures but there was considerable overlap in the range of temperatures that the two species occupied during preference trials. When matched for size, *G. pulex* had a greater leaf shredding efficiency than *D. villosus*, suggesting that invasion and subsequent displacement of the native amphipod will result in reduced ecosystem functioning. However, *D. villosus* is an inherently larger species and interspecific variation in shredding was reduced when animals of a representative size range were compared. *D. villosus* shredding rates increased at a faster rate than *G. pulex* with increasing temperature suggesting that climate change may offset some of the reduction in function. *D. villosus*, but not *G. pulex*, showed evidence of an ability to select those temperatures at which its shredding rate was maximised, and the activation energy for shredding in *D. villosus* was more similar to predictions from metabolic theory. While per capita and mass-corrected shredding rates were lower in the invasive *D. villosus* than the native *G. pulex*, our study provides novel insights in to how the interactive effects of metabolic function, body size, behavioural thermoregulation, and density produce antagonistic effects between anthropogenic stressors.

**Electronic supplementary material:**

The online version of this article (doi:10.1007/s00442-016-3796-x) contains supplementary material, which is available to authorized users.

## Introduction

Biological invasions are a widespread and significant component of human-induced global environmental change, and are having a major impact on the Earth’s ecosystems (Simberloff et al. 2013; Dunn and Hatcher 2015). Invasions also impact world economies, with financial costs reaching over $120 billion per year in the United States (Pimentel et al. 2005) and €12bn per year in Europe (Altmayer 2015). The current rate of alien species introductions is unprecedented, due mainly to globalisation and growth in the volume of trade and tourism (Anderson et al. 2015). These effects make urgent the need to generate a better understanding of the mechanisms that underpin the impacts of invasive species on native species and recipient ecosystems, and how those invasions might interact with other anthropogenic stressors. Invasions by alien species are increasingly being recognised as one of the major threats to biodiversity and ecosystem functioning in freshwater ecosystems (Strayer and Dudgeon 2010). Invasive species can have a variety of effects on the structure of recipient freshwater communities, such as displacing native species (Dick et al. 2002) and altering the diversity and abundance of macroinvertebrate assemblages (Ricciardi 2001). These direct effects, and their underlying mechanisms such as predation and competition, are relatively easy to identify (MacNeil and Platvoet 2005). While the consequences of invasions for ecosystem functioning are less readily understood, research in this area is increasing and there are well-described case studies, such as *Dreissena polymorpha* in the Hudson river (Strayer et al. 1999) and *Corbicula fluminea* in the Plata river (Sousa et al. 2008), that have shed light on how freshwater invaders can dramatically affect ecosystem processes (Strayer 2010).

In both terrestrial and freshwater habitats, macroinvertebrates influence whole ecosystem functioning by accelerating detritus decomposition, and by releasing bound nutrients through their feeding activities and burrowing behaviours (Wallace and Webster 1996; Covich et al. 1999). In freshwater food webs, energy flows from leaf-litter processing are enhanced significantly by shredder consumption, particle fragmentation, and faeces production which convert coarse particulate organic matter (CPOM; organic material >1 mm diameter) into fine particulate organic matter (FPOM; 50 µm–1 mm) (Vannote et al. 1980; Graça 2001; Truhlar et al. 2014). This process makes allochthonous energy inputs accessible to invertebrates that feed directly on FPOM, facilitating trophic energy transfer (Vannote et al. 1980; Graça et al. 2001; MacNeil et al. 2011). Functionally, freshwater amphipods (Crustacea) play significant roles as shredders exerting strong control over the rate of leaf processing (Newman 1990; Navel et al. 2010; Truhlar et al. 2014). Alterations to amphipod assemblage composition can therefore have major consequences for aquatic ecosystem functioning (Piscart et al. 2011).

When introduced to a new area, invasive amphipods often displace their native counterparts due to competition for resources or direct predation pressure (Piscart et al. 2009; Truhlar et al. 2014). This process of displacement has been observed with the Ponto–Caspian amphipod *Dikerogammarus villosus* (Sowinsky, 1894), which has replaced or disrupted the distribution of many resident amphipod species, including previously successful invaders, at numerous sites across Europe (Rewicz et al. 2014). Known as the ‘killer shrimp’ due to its predatory nature, *D. villosus* is a highly voracious, omnivorous, and physiologically tolerant species (Rewicz et al. 2014). It is capable of surviving in ship ballast water promoting its dispersal (Bruijs et al. 2001), and is regarded as one of the worst invasive species in Europe in terms of its negative impact on the functioning and biodiversity of invaded ecosystems (DAISIE 2009). It is expected to expand its range and eventually reach North America (Ricciardi and Rasmussen 1998). In September 2010, *D. villosus* was recorded outside mainland Europe for the first time, in a reservoir called Grafham Water in the UK (MacNeil et al. 2010), and has since established in other parts of England and Wales (MacNeil et al. 2012). Its introduction has already led to community-level changes at invaded sites, including the displacement of the native amphipod *Gammarus pulex* (Linnaeus, 1758) (Madgwick and Aldridge 2011; Truhlar et al. 2014). Previous research into how this invasion may affect ecosystem functioning in freshwaters has indicated that *D. villosus* has a lower leaf shredding efficiency than other amphipod species, including the native *G. pulex* (MacNeil et al. 2011; Jourdan et al. 2016). Consequently, the introduction of *D. villosus* may threaten the fundamental role played by native macroinvertebrate shredders in determining energy flow in these invaded ecosystems (MacNeil et al. 2011).

Life history traits of *D. villosus*, such as early sexual maturity, large reproductive capacity, and high growth rates (Pöckl 2009), as well as its predatory capabilities combined with an omnivorous nature (Dick et al. 2002; Rewicz et al. 2014) confer a large competitive advantage over many other amphipods (Rewicz et al. 2014). For poikilothermic animals such as *D. villosus* and *G. pulex*, the temperature of the surroundings strongly modulates their performance, by driving variation in metabolic rate (Brown et al. 2004; Maazouzi et al. 2011). Increasing metabolic rate with temperature necessarily drives enhanced consumption, and metabolic theory of ecology (MTE) predicts that the activation energy of these consumer-resource interactions should vary around 0.60–0.70 eV similar to those of the underlying biochemical reactions of individual metabolism (e.g., Brown et al. 2004). Deviations from these predictions may provide unique insights into the linkages between biodiversity and ecosystem functioning (e.g., Yvon-Durocher and Allen 2012; Perkins et al. 2015), but studies marrying the functional effects of invaders and native species with metabolic theory have yet to be undertaken.

Behavioural studies on the thermal avoidance and preference of crustaceans have indicated that they exhibit distinct temperature preferences and their thermosensitivity may be in the range of 0.2–2 °C (Lagerspetz and Vainio 2006; González et al. 2010). Devin et al. (2003) demonstrated that *D. villosus* and *G. pulex* prefer similar substratum types, and that the spatial niches of these two species overlap significantly. If these amphipods also demonstrate preferences for similar thermal ranges, then this could further promote direct competition between the two, and increase the threat of the displacement of the native *G. pulex*.

This study investigated the thermal preferences and leaf shredding efficiencies of both the invasive *D. villosus* and the native *G. pulex*, to better understand the combined impacts that species invasion and warming could have on ecosystem functioning in freshwater habitats. This study specifically aimed to test the following predictions concerning our study system: (1) *D. villosus*, characteristic of the eurythermic Ponto–Caspian species, exhibits a broader thermal preference and greater preference for higher temperatures than *G. pulex*; (2) leaf shredding efficiencies for both species increase with temperature in line with predictions of MTE, but are overall higher for *G. pulex* due to its greater preference for plant-based food sources; and (3) both species select temperatures at which they perform optimally. This study provides a comprehensive investigation into the thermal biology of an invasive species relative to a displaced native species, which provides the basis for understanding better the complexity of impacts that both climate change and biological invasions will have on freshwater ecosystem functioning.

## Methods

### Collection and maintenance of animals

Test animals were collected during June and July 2015 through standard sweep sampling, with *D. villosus* collected from Grafham Water in Cambridgeshire (52°18′N; 0°19′W) and *G. pulex* collected from a small stream adjacent to Meanwood Beck in Yorkshire (53°50′N; 1°35′W). Air and water temperature data suggest minimal differences between the sites (Fig. S1). Each species was maintained separately in the laboratory in aerated tanks (30 × 18 × 15 cm) filled with dechlorinated aged tap water at 15 °C under a 16:8 lighting regime. Shelter was provided in the form of gravel and pebbles (Bruijs et al. 2001). Leaves of naturally conditioned alder (*Alnus glutinosa*) and sycamore (*Acer pseudoplatanus*) were provided as a food source, and air stones provided smooth water movement and sufficient dissolved oxygen concentrations (Kley et al. 2009).

### Thermal preference experiments

We used the ‘acute’ method to derive thermal preferenda for each species (Reynolds and Casterlin 1979), whereby three different acclimation temperatures were used (5, 15, or 20 °C) for a 4-day period prior to a 135 min testing phase in a thermal gradient. Temperature selection behaviour was examined using four toroidal (annular) thermal gradient tracks (Fig. S2) modified from Kivivuori and Lagerspetz (1990). Each track was 120 cm (L) × 11 cm (W). An ice bath was used to cool one end of the track, with a water bath heating the opposite end. The resultant water temperature gradient ranged from 4 to 24 °C (Fig. S3, raw data in Table S1), measured using 16 evenly spaced digital thermometers (Avax DT-1) The bottom of the apparatus was lined with a thin layer of gravel (ca. 2 mm particle size), and water depth was 2 cm to prevent thermal stratification of the water column. All tracks were illuminated evenly to prevent dark-seeking behaviour.

For each acclimation temperature, 30 *G. pulex* and 30 *D. villosus* were introduced to the gradient apparatus in pairs to determine thermal preferenda. A 1:1 male:female ratio was used, and individuals were selected that represented the full range of body sizes for adults. After the experiment, amphipods were preserved in 70% ethanol, weighed, and then photographed to determine body length using ImageJ software. As both body length (Lewis et al. 2010) and mass (Navel et al. 2010) have been used as predictors of energetic demands for amphipods, these two correlated metrics were combined into a single index of amphipod ‘body size’ using principal component analysis (Truhlar et al. 2014). Individuals were introduced to the section of the gradient corresponding to their acclimation temperature to reduce stress caused to the animal, and were left for a 30 min period initially to reduce the impact of handling on behaviour. The water temperature of the position of each individual within the track was then recorded every 3 min for a period of 45 min. To ensure that both species showed no preference for any particular position in the track, control experiments were carried out using six animals from each species and recording amphipod locations every 2 min when the water was a uniform temperature of 20 °C. A concern arising from test animals being introduced in pairs is that they may interact socially so cannot be treated as independent individuals (Karlsson et al. 1984); however pilot data comparing individual and paired animals suggested that grouping did not affect thermal behaviour in the gradients.

### Leaf shredding experiments

Leaves of the sycamore tree (*A. pseudoplatanus*) were provided as the food source, as this tree is common at the collection sites of both species and its leaves have been shown to be highly palatable to amphipods (MacNeil and Platvoet 2005). Leaves were conditioned in stream water for two weeks at 15 °C, which allowed the leaching of soluble components, softening, and encouraged fungal growth (Bloor 2011). Leaves were cut into 6 mm-diameter leaf discs using a cork-borer, with midribs and any obvious infected areas avoided, and these were then air-dried, sorted into batches of five, and weighed (leaf batch air-dry mass = 16.00 ± 3.27 mg, *n* = 320).

*Dikerogammarus villosus* are an inherently larger species than *G. pulex* (animals used in this study were: *G. pulex* length = 12.10 ± 0.10 mm, range 7.35–17.86 mm; *D. villosus* length = 15.89 ± 0.18 mm, range 9.13–25.77 mm). Therefore, all shredding experiments were conducted with the full range of body sizes, but at least half of all replicates used size-matched individuals to avoid the confounding effects of variation in body size and reproductive cycle (Truhlar et al. 2014). Full data on the sizes of all specimens can be found in Table S4 along with the results of the experiment.

Eight experimental temperatures (5, 8, 10, 12.5, 15.5, 17.5, 20, and 22.5 °C, chosen to cover the range of UK river temperatures, Garner et al. 2014) were used to assess the effect of temperature on shredding efficiency for both species of amphipod. All trials were subject to a uniform photoperiod of 16:8, and the water temperature at each experimental temperature was recorded for the duration of the trials using Tinytag Plus 2 TGP-4017 dataloggers (Gemini Data Loggers). Each species was tested separately with 10 replicates of size-matched individuals and 10 replicates containing amphipods covering the remaining range of each species’ body sizes, giving 8 temperature treatments, 2 species treatments, and 2 size treatments, each replicated 10 times for 320 replicates in total. Each replicate was established in a plastic container [8 cm (*ø*) × 7 cm (D)] filled with dechlorinated tap water along with three clear glass pebbles (2-cm diameter) to provide animals a retreat whilst still permitting observation (MacNeil and Platvoet 2005). Two animals were placed in each pot and were subjected to a 24 h starvation period at their experimental temperature prior to testing. Each replicate involved two animals for two reasons: (1) mortality was relatively high at higher temperatures and so multiple animals gave a higher chance of the 72 h incubation yielding at least one animal alive at the end, and (2) shredding rates were measured over a relatively short period and so the combined shredding of two animals gave a stronger signal. At the start of each trial, a pre-weighed batch of five leaf discs was added to each pot. Each trial lasted for 72 h, with amphipod deaths recorded every 24 h and dead animals being removed (Truhlar et al. 2014). At the end of each experiment, the animals were weighed and photographed for their body length to be measured using ImageJ software. Animals were retained for 3 days post-experiment, and any that moulted was removed from subsequent data analyses (Paterson et al. 2015). Remaining leaf discs were dried for 24 h at 90 °C and weighed. Control pots established at each temperature consisted of amphipod-free pots with only leaf discs added.

### Data analysis

#### Thermal preferences

In the control experiment with the gradient apparatus held at 20 °C, animal locations were classified into regions of the track of length 10 cm and Chi-squared tests were used to assess preference. For each species, 30 recordings were taken of 6 specimens, giving 180 recordings for each species. In the main experiment with thermal gradient, the median selected temperature during the period of observation was calculated to avoid pseudoreplication and provide a measure of preference for each individual (Karlsson et al. 1984). Median preferenda were then examined with respect to amphipod species, acclimation temperature, body size, and sex in linear mixed effects model using the lme4 (Bates et al. 2015) and lmerTest (Kuznetsova et al. 2015) packages in R v3.2.0 (R Core Team 2015), with time of experiment and track as random effects, and slopes and intercepts allowed to vary at random. Following data transformation to account for leptokurtosis in the residuals, model selection carried out on this global model using the dredge function in the MuMIn package (Barton 2015) in R, and model averaging was used to take the weighted averages of the parameters of those models with ∆AIC < 4, providing a final mixed effects model of ‘Species + Acclimation temperature + Species × Acclimation temperature’ with ‘experimental track’ and ‘time of run’ accounted for as random effects.

At each acclimation temperature, the average acute thermal preferendum for each species was calculated as the mean of the selected temperatures (Reiser et al. 2014). The final thermal preferendum derived by the acute method is defined as that temperature where preference equals acclimation temperature (Fry 1947). Therefore, to determine this value for each species, acute thermal preferenda were plotted with a 1:1 regression line (where response temperatures and acclimation temperatures are equal), and the final thermal preferendum of each species was calculated as the point of intersection between this line of equality and the trend line describing the acute thermal preferenda (Reynolds and Casterlin 1979).

#### Leaf shredding

Leaf shredding efficiency was measured as the dry mass of leaf consumed per amphipod/day (Truhlar et al. 2014). To account for the effects of amphipod deaths, the leaf mass consumed in each replicate was standardised by the number of amphipod days in that replicate, where amphipod days was equal to the number of surviving amphipods on a given day summed over all 3 days of the experiment. To compare shredding efficiency between species, size-matched male amphipods were used. The two correlated metrics of wet mass and body length were combined using PCA into a single index of ‘body size’. The species scores from PC1 were then analysed using one-way ANOVA to confirm successful size-matching. Leaf shredding efficiency was then examined with respect to amphipod species and temperature in a two-way ANOVA. Non-significant terms were removed via stepwise deletion. Data were then pooled to incorporate the results from the full size ranges for both species, and leaf shredding efficiency was again examined with respect to species and temperature in a two-way ANOVA. Post-hoc testing for both the above models was conducted using Tukey’s HSD tests.

For each experimental temperature treatment, water temperature was converted to 1/*kT*_c_ − 1/*kT* where *k* is the Boltzmann constant (8.62 × 10^−5^ eV K^−1^), *T* is temperature in °K (Yvon-Durocher et al. 2012), and c denotes the intercept temperature for 15 °C (288.15 °K); higher values of this standardised variable therefore relate to higher water temperature. Temperatures were plotted against ln transformed leaf shredding efficiency and relationships determined using linear regression in R2.14.0. Regression multipliers provide estimates of the activation energy of leaf shredding efficiency. ANCOVA was used to assess whether the relationship between temperature and leaf shredding differed between the two species.

#### Thermal preference versus shredding performance

Temperature zones (ranging 1 °C either side) were assigned to each experimental temperature tested in the shredding trials (i.e., the zone for 15.5 °C temperature would be 14.5–16.5 °C). Then, for each species, the relationship between habitat use (mean number of position records per individual in a temperature zone for 15 °C acclimated animals) and functional performance (mean leaf mass consumed per individual in the corresponding temperature zone) was examined using orthogonal non-linear least squares regression (ONLS, as both variables were measured with error) to test for an asymptote that would indicate that the amphipod species selected temperatures at which they performed optimally. Specifically, the model fitted using the ONLS approach was ‘shredding ~ *α* + *β*/habitat use’. The measure of functional performance was taken as mean leaf mass consumed per individual over 72-h, as opposed to mean shredding efficiency, as this measure partially accounted for the increased mortality rates that were observed with increasing temperatures for both species.

## Results

### Thermal preference experiments

**Fig. 1:**
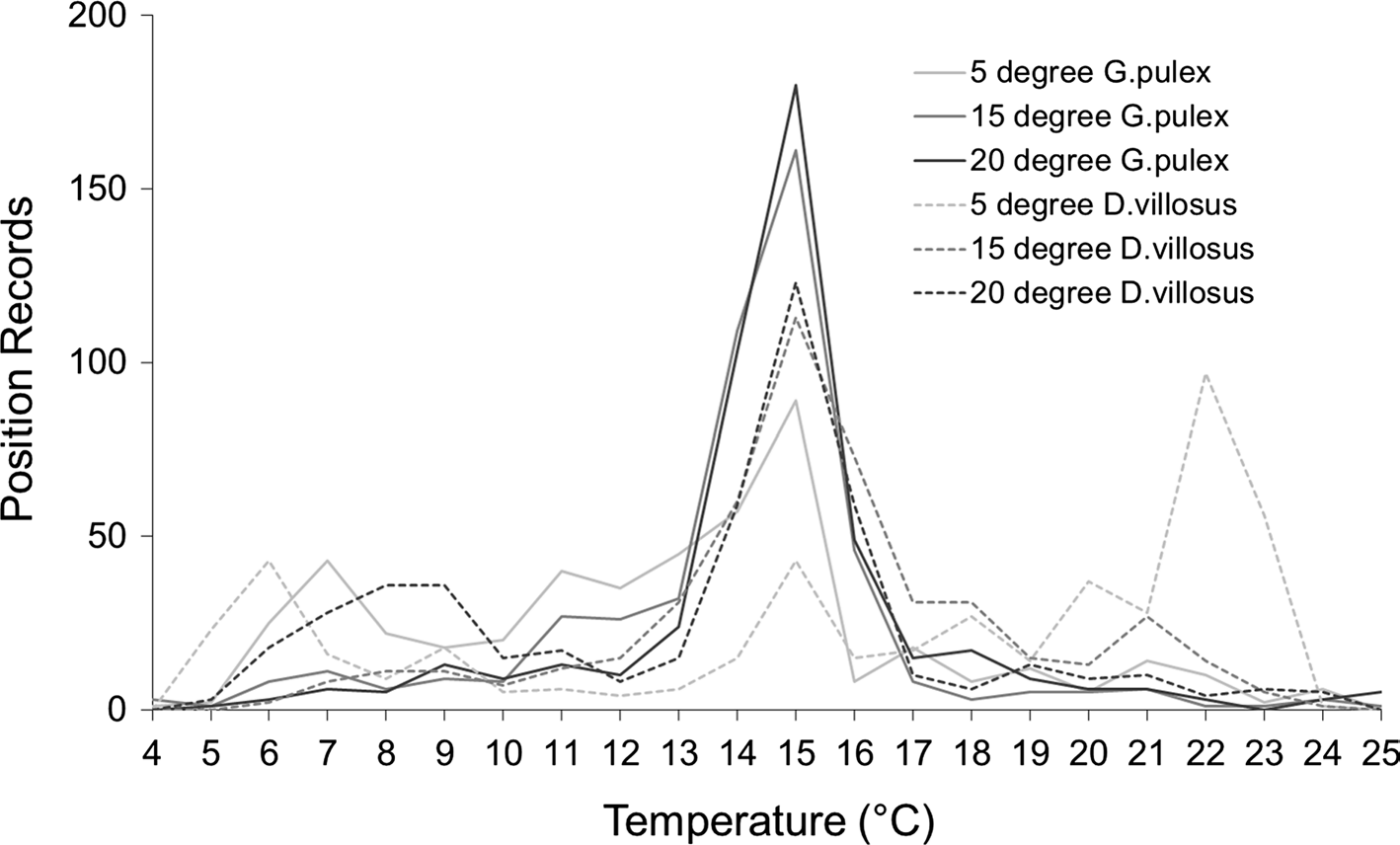
Position records of *G. pulex* (90 animals) and *D. villosus* (90 animals) in the temperature gradients. Prior to the experiments, *G. pulex* (*solid lines*) and *D. villosus* (*dashed lines*) individuals were acclimated to either 5 °C (*light grey*), 15 °C (*medium grey*), or 20 °C (*dark grey*)

**Fig. 2:**
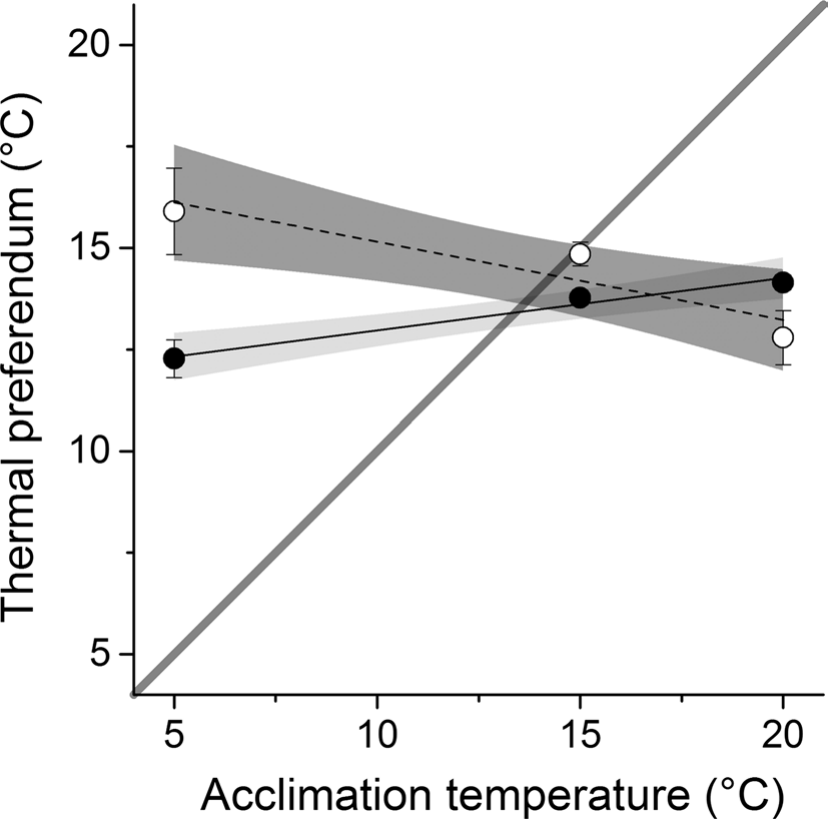
Acute thermal preference of *G. pulex* (*filled symbols*, *solid line*, *light grey* area denotes 95% CI) and *D. villosus* (*open symbols*, *dashed line*, *dark shaded area* denotes 95% CI). *Error bars* denote ±1 SE. *Grey line* indicates the line of equality (i.e. where acclimation temperature and preferred temperature are equal). The point of intersection between these *lines* indicates the final thermal preferendum for each species

At all acclimation temperatures, both *G. pulex* and *D. villosus* displayed a distinct preference for a narrow temperature range between 13 and 16 °C (Fig. 1, raw data can be found in Table S3). From the linear mixed effects model, the acute thermal preferences differed significantly between the two species, with species featuring in all top models (Table 1) and being statistically significant in the top model (Table 2). *D. villosus* preferred higher temperatures to *G. pulex*. Acclimation temperature also had a significant effect on thermal preferences, and interestingly, there was a significant difference between the species × acclimation temperature interaction, indicating that the effect of acclimation temperature on preference temperature was different for each amphipod species. Specifically, as acclimation temperature increased the preference temperature of *G. pulex* also increased, but this pattern was reversed for *D. villosus*, with its preference temperature decreasing with increasing acclimation temperature (Fig. 2). Based on the thermal preferenda derived for the three acclimation temperatures (Fig. 2), the final thermal preferendum using the acute method was calculated at 13.4 °C for *G. pulex* and 14.3 °C for *D. villosus.* Neither *G. pulex* (*χ* ^2^ = 6.93, *df* = 11, *p* = 0.805) nor *D. villosus* (*χ* ^2^ = 15.13, *df* = 11, *p* = 0.176) showed a preference for any particular section of the track when the water temperature was held at a uniform temperature of 20 °C (i.e., control conditions, Fig. S4, full data can be found in Table S2).

**Table 1:** Subset of linear mixed effects models with ∆AIC < 4 that describe thermal preferences in two species of amphipods, *G. pulex* and *D. villosus*

Model	*df*	AICc	ΔAIC	W_i_
Species + Acc.Temp + Species × Acc.Temp	7	555.9	0.00	0.660
Species	5	557.9	1.98	0.246
Species + Acc.Temp + Sex + Species × Acc.Temp	8	559.8	3.89	0.094

All models include ‘experimental track’ and ‘time of run’ as random effects. “Acc.Temp” = acclimation temperature

**Table 2:** Results of the final minimum linear mixed effects model (Sp + Acc.Temp + Sp × Acc.Temp) for temperature preference, with ‘experimental track’ and ‘time of run’ as random effects

Parameter	Estimate	SE	*T*	*i*
Intercept	0.974	0.261	3.736	<0.001
Species	−2.028	0.369	−5.500	<0.001
Acc.Temp	−0.057	0.018	−3.231	0.001
Species × Acc.Temp	0.111	0.025	4.419	<0.001

“Acc.Temp” = acclimation temperature

### Leaf shredding experiments

#### Leaf shredding by size-matched individuals

**Fig. 3:**
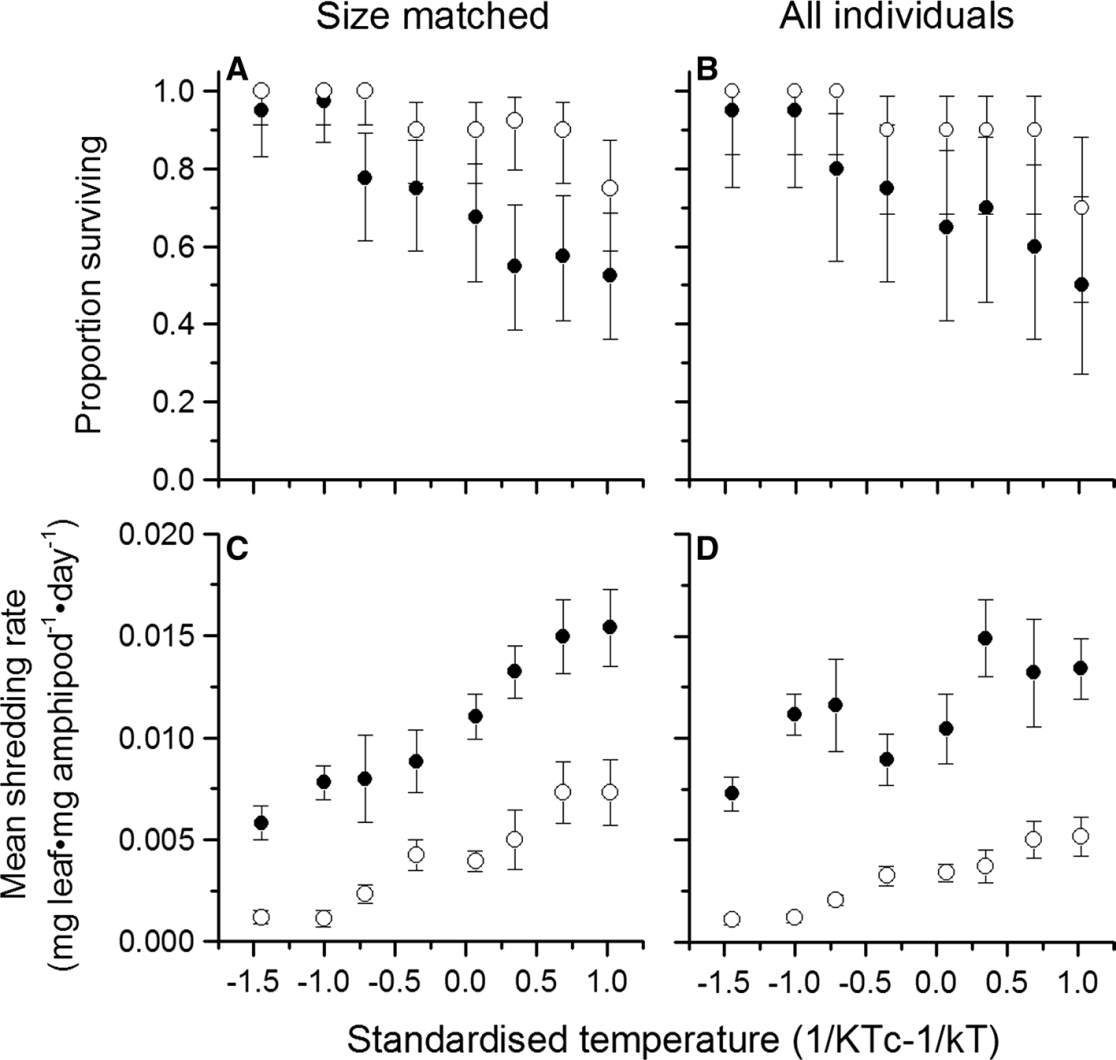
Relationship between temperature and **a** survival in size-matched amphipods, **b** survival in the whole sample of amphipods, **c** shredding rates for size-matched amphipods, and **d** shredding rates for the whole sample of amphipods. Points are mean values (±1 SE for shredding; 95% CI for survival), for *G. pulex* (*filled symbols*) and *D. villosus* (*open circles*)

Two-way ANOVA showed that leaf shredding efficiency was significantly affected by both amphipod species and temperature (Table 3). The interaction between these two variables was not significant, indicating that both species responded the same way to increasing temperature with respect to their shredding efficiencies, and this interaction was thus removed from the model. *G. pulex* displayed a significantly greater leaf shredding efficiency than *D. villosus*, and leaf shredding efficiency increased with temperature for both species (Fig. 3, raw data can be found in Table S4). The observed activation energy of shredding efficiency was 0.83 eV for *D. villosus* although the theoretically predicted range (0.6–0.7 eV) fell within the 95% confidence intervals of the regression (0.56–1.10 eV). In contrast, *G. pulex* estimates were outside of MTE predictions (0.40 eV; 95% CI 0.46–0.34). ANCOVA showed a significant effect of species identity on the relationship between temperature and shredding (*F*_3,12_ = 14.19, *p* = 0.003). PC1 explained 93.8% of the variance of body length and wet mass in the amphipods, making it a highly reliable index of overall body size. The one-way ANOVA of PC1 scores with respect to species for the size-matched individuals showed no significant difference (*F*_1,319_ = 0.708, *p* = 0.401) between the body sizes of *G. pulex* (length = 13.29 ± 1.46 cm, weight = 0.037 ± 0.011 g) and *D. villosus* (length = 13.37 ± 1.60 cm, weight = 0.039 ± 0.013 g), therefore the size-matching was considered successful. Leaf discs in the control aquaria (no animals present) had a negligible mass loss (<2% of the mass of initial discs added) over the duration of the experiment, and so loss of leaf mass due to microbial breakdown or leaching was discounted (MacNeil et al. 2011).

**Table 3:** Results of two-way analysis of variance testing the effects of species and temperature on overall leaf shredding efficiency in size-matched individuals and for all individuals pooled

Size classes	Source	*df*	*F*	*p*
Size-matched	Species	1148	169.580	<0.001
	Temperature	1148	92.386	<0.001
	Species × temperature	1148	0.095	0.759
All individuals	Species	1302	93.551	<0.001
	Temperature	1302	57.833	<0.001
	Species × temperature	1302	3.917	0.049

#### Leaf shredding across all size classes

The large *D. villosus* consumed more leaf mass per amphipod day over all temperatures compared to smaller conspecifics, and the large-sized *G. pulex* only consumed more at temperatures of 12.5 °C and above (Fig. 3). Similar to the results for size-matched individuals, the two-way ANOVA found that leaf shredding efficiency was significantly affected by both amphipod species and temperature. However, in contrast to results from size-matched animals, the interaction between these two variables was significant indicating that the two species differed in the nature of the relationship between shredding and temperature (Table 3).

When all sizes of individuals were taken into consideration, *G. pulex* still displayed a significantly greater leaf shredding efficiency than *D. villosus* (Fig. 3). Leaf shredding efficiency increased with temperature for *D. villosus*. However, this tendency was much less pronounced for *G. pulex* owing mainly to the reduced leaf shredding efficiency of smaller individuals at higher temperatures. Increasing temperatures had a greater effect on the mortality rate of *G. pulex* than on *D. villosus* (Fig. 3). The observed activation energy of shredding efficiency for *D. villosus* was within the range predicted by MTE (0.68 ± 0.20 eV, Table 4). In contrast, *G. pulex* estimates were outside of MTE predictions (0.21 eV; 95% CI 0.03–0.39). ANCOVA showed a significant effect of species identity on the relationship between temperature and shredding (*F*_3,12_ = 18.82, *p* < 0.001), as was seen for the raw shredding data (Table 3).

**Table 4:** Regression parameters for ln mean shredding rates as a function of water temperature [1/*kT* (15 °C) − 1/*kT*)

Species	Data type	Intercept (±95% CI)	Multiplier (±95% CI)	*p*	*R* ^2^
*G. pulex*	Size-matched	−4.53 (±0.05)	0.40 (±0.06)	<0.001	0.98
	All data	−4.46 (±0.15)	0.21 (±0.18)	0.03	0.57
*D. villosus*	Size-matched	−5.57 (±0.22)	0.83 (±0.27)	<0.001	0.91
	All data	−5.80 (±0.16)	0.68 (±0.20)	<0.001	0.92

### Thermal preference versus shredding performance

**Fig. 4:**
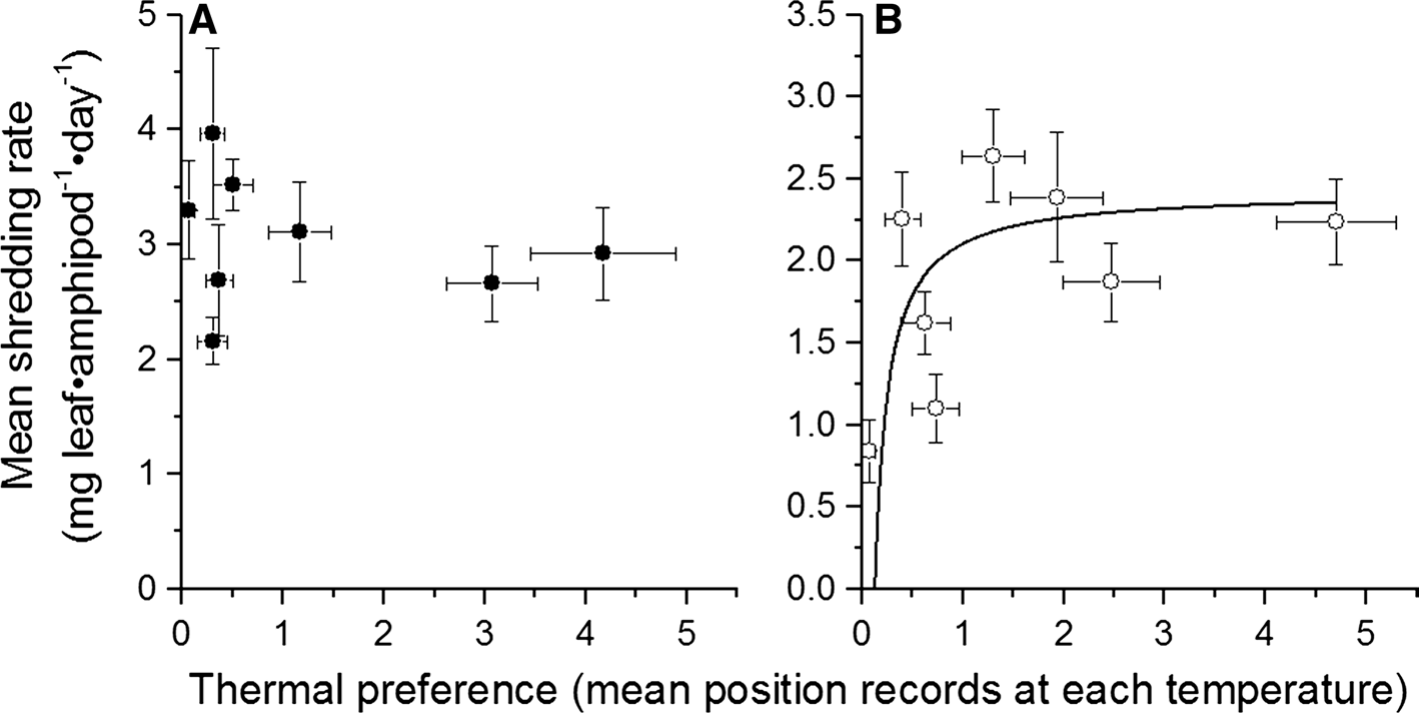
Relationship between habitat use and functional performance for **a** *G. pulex* and **b** *D. villosus*. Fitted line in **b** is the result of an orthogonal non-linear least squares regression that takes into account error in both *x* and *y* variables (see text for details). *Error bars* denote ±1 SE for both variables

ONLS regressions showed that there was no relationship between our measure of thermal preference (the time over which a particular thermal microclimate was used) and the measure of performance (per capita leaf shredding) in *G. pulex* (*t* = 0.830, *p* = 0.438), but that the preference did explain the increase to asymptote in *D. villosus* (*t* = −2.915, *p* = 0.027; Fig. 4).

**Fig. 5:**
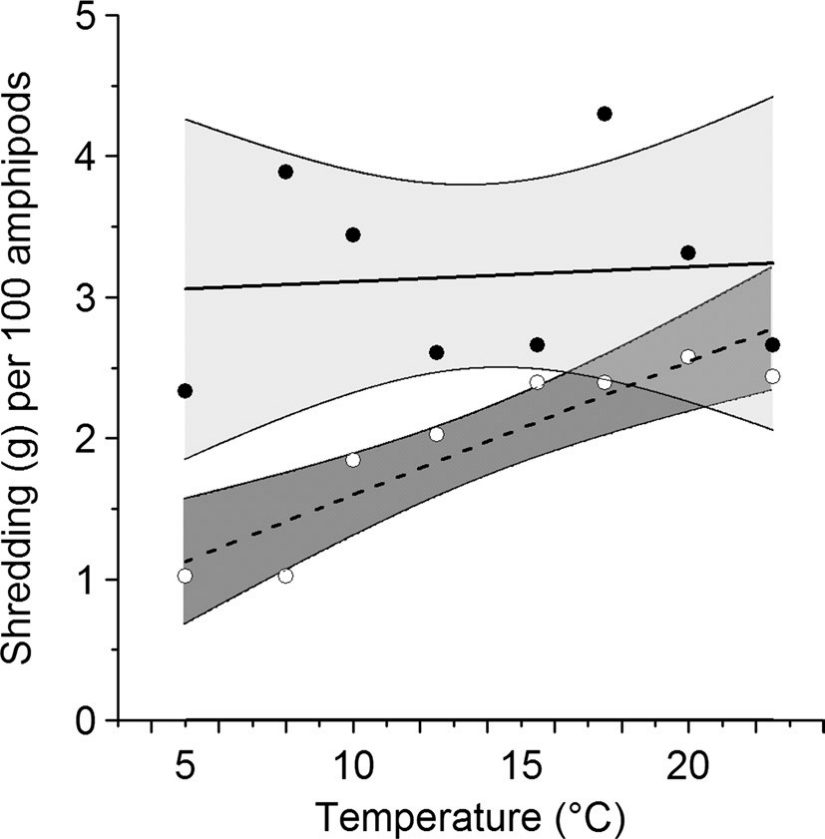
Predictions of shredding (g leaf mass per 72 h) in theoretical populations of 100 *G. pulex* (*filled symbols*, *solid line*, *light grey area* denotes 95% CI) and 100 *D. villosus* (*open symbols*, *dashed line*, *dark shaded area* denotes 95% CI) over a 72 h period. Shredding capacity is the product of mass-specific shredding rate over 72 h and the mean mass of each species (30.5 mg in *G. pulex*, 68.2 mg in *D. villosus*), multiplied by the survival rate at that temperature. Per capita post-mortality rates are multiplied by 100 to give an estimated mortality for the hypothetical population

Combining the shredding rates, mortality rates, and body size measurements allows prediction of the potential consequences of replacement of *G. pulex* by *D. villosus* (Fig. 5). Population-level shredding capacity shows no relationship with temperature in *G. pulex* (*r* = 0.091, *p* = 0.830), demonstrating that increased mortality at higher temperatures cancels-out any increase in shredding efficiency. However, population-level shredding in *D. villosus* continues to increase approximately linearly with temperature (*r* = 0.913, *p* = 0.002; Fig. 5). The regression lines suggest *G. pulex* shreds 200% more leaf matter at 5 °C but only 20% more at 22.5 °C, hence replacement by *D. villosus* is predicted to result in smaller declines in resource processing at warmer temperatures.

## Discussion

Animal invasions are being recognised increasingly as a major threat to biodiversity and ecosystem function in freshwater ecosystems (Simberloff et al. 2013). Here, we have demonstrated that the invasive amphipod *D. villosus* shreds less leaf mass than the native species *G. pulex*. However, we show that any decline in ecosystem function following replacement of the native by the invasive is likely to be offset by the greater size of the invasive species, climate-induced warming of the aquatic environment, and the ability of the invasive species to select those microclimates that optimise its performance which is absent from the native species.

### Thermal preference experiments

The results from this study clearly demonstrate thermal preference behaviour in both *D. villosus* and *G. pulex*, consistent with previous work on crustaceans (Lagerspetz and Vainio 2006; González et al. 2010; Reiser et al. 2014). Neither body size nor sex had a significant effect on temperature preference, indicating that the final thermal preferenda derived from this study appear to be representative of all individuals of these species, at least individuals from the populations where we collected specimens. The thermal preference of a species depends on its evolutionary thermal history (Lagerspetz and Vainio 2006), which may account for the slightly higher thermal preferendum found for *D. villosus*, as it is native to the Ponto–Caspian basin where summer water temperatures may reach 29 °C at its peak (Rewicz et al. 2014). The native and the invasive amphipods spent the majority of their time in similar water temperatures ranging between 13 and 16 °C, suggesting similar thermal niches and therefore a high potential for direct competition (McMahon et al. 2006). Previous research has shown that when both *G. pulex* and *D. villosus* are present in microcosm and mesocosm experiments, *G. pulex* suffer severe intraguild predation from *D. villosus* with no reciprocal predation observed (Dick et al. 2002; MacNeil et al. 2011). Field studies have also shown that populations of native *G. pulex* decline after *D. villosus* invasion (Madgwick and Aldridge 2011). Therefore, in invaded ecosystems, direct competition resulting from overlapping thermal niches would likely result in the displacement of *G. pulex* by *D. villosus*.

### Leaf shredding experiments

The invasive *D. villosus* exhibited lower leaf shredding efficiency than the native *G. pulex*, consistent with previous studies (MacNeil et al. 2011; Piscart et al. 2011). In isolation, this observation may lead to the prediction of serious implications for ecosystem functioning in invaded waterways, as a decrease in leaf-litter processing would result in a reduction of FPOM production, consequently reducing energy inputs accessible to other macroinvertebrates and disrupting energy transfer between trophic levels (Vannote et al. 1980; Graça et al. 2001). In contrast to these results, Truhlar et al. (2014) observed that *D. villosus* was significantly more efficient at shredding leaves than *G. pulex* when experiments were carried out at 25 °C. Potential explanations for this difference are that the experimental temperatures in the present study only reached 22.5 °C, while 25 °C may have greater associated costs for *G. pulex* than *D. villosus*, and that Truhlar et al. (2014) used unconditioned *Salix* leaves as the food source in their shredding experiments. The present study used conditioned *Acer* leaves and *D. villosus* may be able to utilise unconditioned leaf more effectively than *G. pulex* (Truhlar et al. 2014).

Leaf shredding efficiency of both *G. pulex* and *D. villosus* increased significantly with temperature but MTE-predicted activation energies applied only to *D. villosus* and not to *G. pulex*. This poses the question of why this is the case and what are the wider implications? *G. pulex* is a cool-adapted species and is seemingly unable to maintain its rate of shredding at higher temperatures, contributing to the lower activation energy and enhanced mortality. One potential reason for its elevated consumption across all temperatures (and confirmed by the higher intercept from the regressions) is that the nutrient stoichiometry of sycamore is inadequate for *G. pulex*; hence it has to consume more leaf to meet its metabolic demands (c.f. Tuchman et al. 2002). This would suggest *G. pulex* to be more selective in terms of detrital matter than *D. villosus*. Further experiments with other types of leaf litter are needed to test this hypothesis, but sycamore has previously been shown to underpin slower growth rates amongst *G. pulex* compared with elm leaf (Sutcliffe et al. 1981). An increase in detrital leaf shredding by *D. villosus* is likely to have wider implications within aquatic communities, for example by increasing available nutrients after leaf decomposition and thus potentially increasing primary productivity. A net result of this interspecific difference in leaf consumption would be more successful invasion by *D. villosus* as it spends less time foraging and feeding, and can allocate more resource to growth and reproduction.

For *G. pulex*, no relationship was found between habitat use and functional performance, however for *D. villosus* there was evidence of a positive relationship, indicating that individuals may spend a greater proportion of their time within thermal limits where they had a greater functional performance: *G. pulex* only spent 8.7% of their time in the temperatures where they performed best, compared to *D. villosus* that spent 29.7% of their time there. This result provides evidence that *D. villosus*, but not the native *G. pulex*, may optimise its performance through selective use of microclimates. Coupled with this was the finding that *D. villosus* had a lower mortality rate than *G. pulex* at every temperature. These eurythermic traits demonstrated by *D. villosus* are common in Ponto–Caspian invaders, which are also commonly euryoecious and euryhaline species tolerant of rapid environmental change (Rewicz et al. 2014). These traits are likely to have contributed to its invasion success in the thermally heterogeneous freshwater environments of Europe. These findings are important in relation to global warming, as not only will temperatures increase over the coming years (UK Met Office 2011), but there will also be an increased variation in daily temperatures (Schar et al. 2004), and this appears to favour the invasive *D. villosus* over the native *G. pulex*.

### Summary

The main findings of this study suggest that invasion by *D. villosus* and the consequent displacement of *G. pulex* will result in reduced leaf decomposition rates due to the lower shredding efficiency of the invader. However, for this system, at least, it appears that the larger size of the invasive species and the effect of environmental warming will partly offset this negative effect through increased resource processing in the invasive species at higher temperatures. Uniquely, this study has shown that the replacement may not impact ecosystem functioning as much as previously thought if other factors enhance the shredding activity of the invasive species, although the higher predatory efficiency of *D. villosus* may reduce overall shredding through predation on other macroinvertebrate shredders (Dodd et al. 2014). Our findings therefore constitute a case of antagonistic stressors (Jackson et al. 2016) and provide new insights into the interactions that link environmental thermal regimes with ecological responses across multiple levels of organisation (i.e., metabolic processes of individuals, populations dynamics of invasive and native species, and ecosystem functioning; cf. Woodward et al. 2010). The wider application of MTE analysis, with respect to invasive species, could prove beneficial in terms of identifying ‘risk’ species during horizon scanning. The results of this study will help predict the possible effect that *D. villosus* will have on freshwater ecosystems as it displaces native species under a warming climate. While estuaries, lakes, and stream outlets represent the current strongholds of *D. villosus*, suitable habitats exist in lower order streams (especially where channelised) and colonisation may be restricted only by stochastic processes (Altermatt et al. 2016), hence further colonisation of headwaters is likely to be a matter of time for this and many other Ponto–Caspian species (Gallardo and Aldridge 2015). Studying and understanding these complex linkages and feedbacks in more detail is vital if ecologists are to deliver more effective modelling of invasion dynamics to inform prevention and mitigation measures.

## Electronic supplementary material

Below is the link to the electronic supplementary material.

Supplementary material 1 (DOCX 645 kb)
