# Supplementary material for: Antagonistic effects of biological invasion and environmental warming on detritus processing in freshwater ecosystems
Source: Oecologia. 2016 Dec 24;183(3):875–86. doi: 10.1007/s00442-016-3796-x (PMC5339318; doi:10.1007/s00442-016-3796-x)
Supplement: Supplementary file 1 — Supplementary material 1 (DOCX 645 kb) [file 442_2016_3796_MOESM1_ESM.docx]

**Antagonistic effects of biological invasion and environmental warming on detritus processing in freshwater ecosystems**

**Supplementary Information**

Daniel Kenna^1^, William N. W. Fincham^1^, Alison M. Dunn^1^, Lee E. Brown^2^, Christopher Hassall^1^

^1^ School of Biology & water@leeds, University of Leeds; ^2^ School of Geography & water@leeds, University of Leeds

Corresponding author:

Email: [c.hassall@leeds.ac.uk](mailto:c.hassall@leeds.ac.uk)

Telephone: +44 113 3435578

Fax: +44 113 343 2835

**Declaration of authorship**: DK, CH, WF, AD and LB conceived the experiment, DK carried out the experiment, DK and CH analysed the data, WF and LB performed the metabolic scaling analysis, and DK, CH, WF, AD and LB wrote the paper.

**Supplementary tables**

Supplementary tables can be found at FigShare at the following link: <https://figshare.com/articles/Kenna_et_al_SI_Tables_xlsx/4254275>

**Table S1.** Temperature at nine points along the temperature gradient measured every 15 minutes over the 45 minute observation period (n=15 observations at each location).

**Table S2.** Control experiments carried out with uniform temperature (20°C) throughout entire track. N=6 specimens of each species used

**Table S3.** Final data for temperature preference experiment - showing all size and weights of individuals and median temperature preferences

**Table S4.** All data from shredding experiments, including animals that were excluded from the analysis due to cannibalism or that moulting (see notes). Sample sizes are n=20 for each species at each temperature.

**
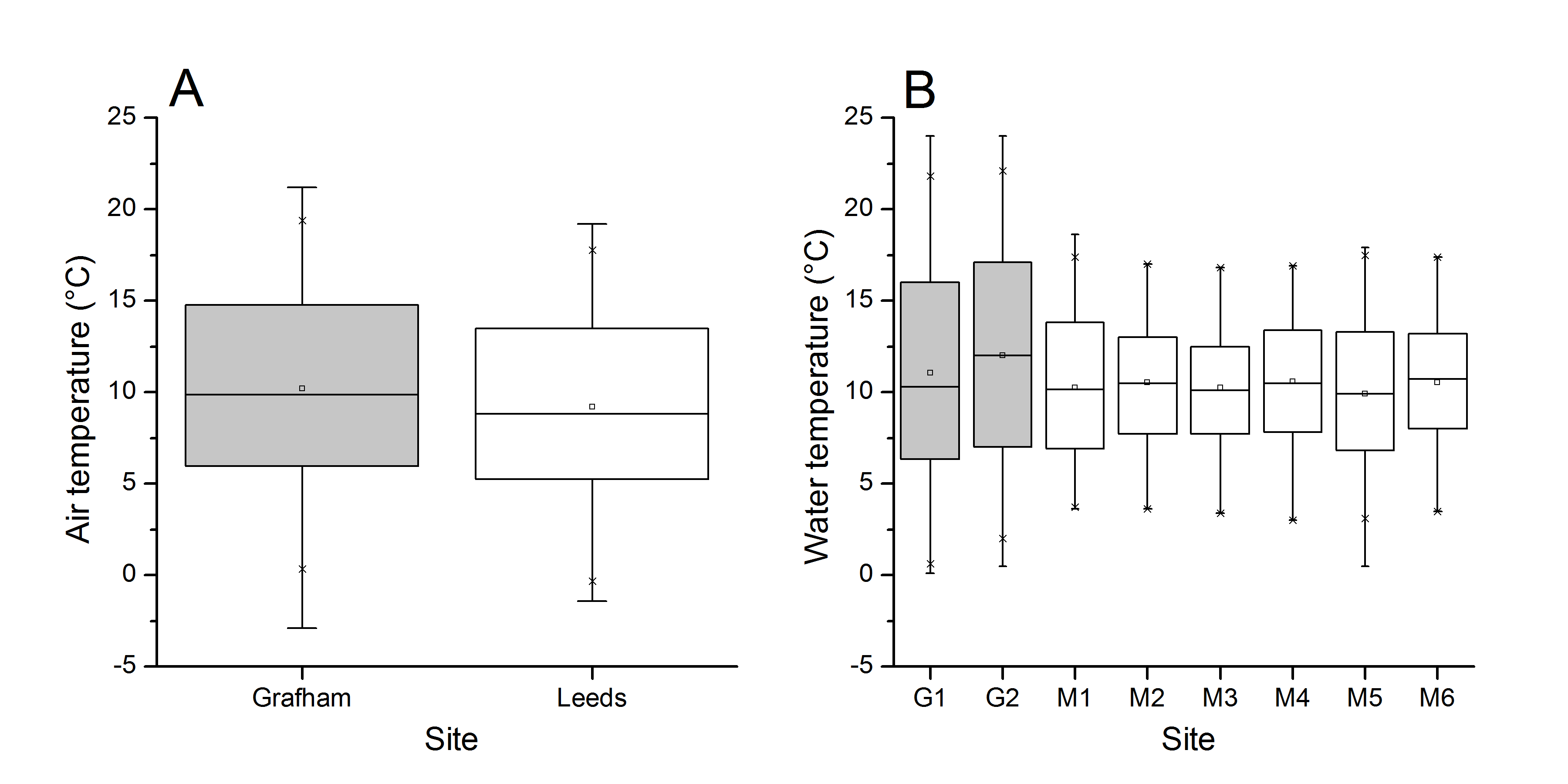
**

Figure S1: To evaluate the thermal environments of the two populations, we compared monthly air temperature records from 1959 to 2015 (684 measurements) at weather stations in Bradford (53.813°N, -1.772°E, 13km from Meanwood Beck) and Cambridge (52.245°N, 0.102°E, 26km from Grafham Water). While mean annual temperature is marginally greater at the Grafham site (10.2°C ± 4.9 SD) than in Leeds (9.2°C ± 4.6), minimum temperatures are in fact lower at Grafham (-0.1°C ± 5.8) than Leeds (0.6°C ± 5.2) due to the more continental climate experienced at that site during the winter (Figure S1A). We then followed-up the air temperature analysis by examining water temperature data from UK Environment Agency spot monitoring databases. While highly heterogeneous in timing and number of samples, the data confirm that the variability in temperature is more than sufficient to swamp any small differences in mean temperature at the two sites (Figure S1B).


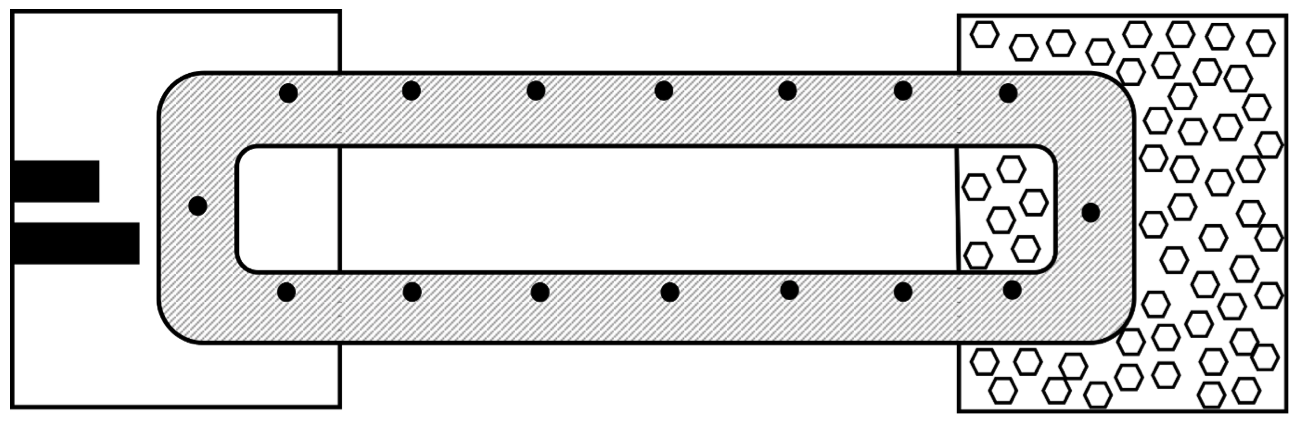


Figure S2: Top view of the toroidal thermal gradient (length 120cm, width of tubing 11cm). The white (to allow easy identification of animals) toroidal PVCu tubing was filled with dechlorinated tap water up to depth of 2cm. The figure shows an external water bath on the left hand side (fitted with two aquarium heaters – 50W and 75W) and an ice bath on the right hand side, in which the toroidal tubing was partly submerged, for the warming and cooling of the ends respectively. The temperature of the gradient was measured by digital thermometers as indicated by black circles. There was a thin layer of white gravel on the bottom of the tubing.


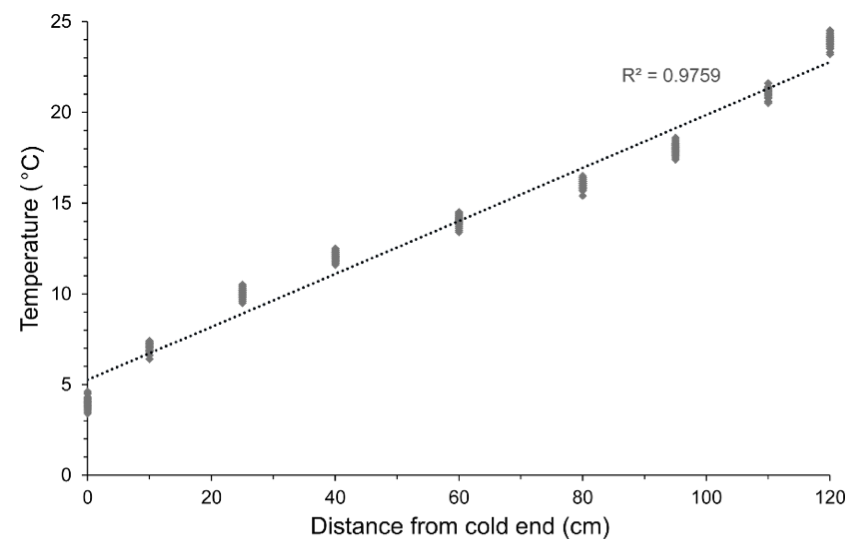


Figure S3: Water temperature recordings in the toroidal gradient tracks were taken every 15 minutes during each 45 minute trial (90 trials in total), and were measured at nine evenly spaced points throughout the track in order to ensure the gradient was being maintained. Temperatures were recorded at both ends of the apparatus, as well as at points 10, 25, 40, 60, 80, 95, and 110cm along from the cold end.


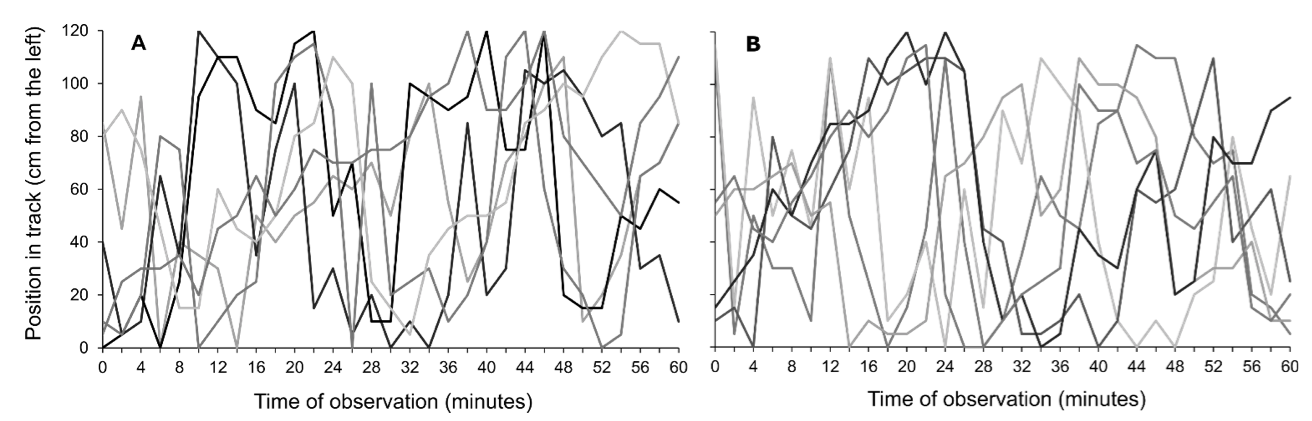


Figure S4: Movements of amphipods in the toroidal track when water temperature was held at a uniform temperature of 20^o^C, with (A) showing movements of *Gammarus pulex* individuals and (B) showing movements of *Dikerogammarus villosus* individuals. For both species, n = 6.
